# Decentralised facility-based training as an alternative model for SLMTA implementation: The Cameroon experience

**DOI:** 10.4102/ajlm.v3i2.231

**Published:** 2014-11-03

**Authors:** Juliana Ndasi, Laura Dimite, Victor Mbome, Charles Awasom, Elive Ngale, Sidney Akuro, Ewane Leonard, Omotayo Bolu, Terence Asong, Patrick Njukeng, Judith Shang

**Affiliations:** 1 Global Health Systems Solutions, Cameroon; 2 US Centers for Disease Control and Prevention (CDC), Cameroon; 3 Buea Regional Hospital, South West Region, Cameroon; 4 Bamenda Regional Hospital, North West Region, Cameroon

## Abstract

**Background:**

The Strengthening Laboratory Management Toward Accreditation (SLMTA) programme is designed to build institutional capacity to help strengthen the tiered laboratory system. Most countries implement the SLMTA three-workshop series using a centralised model, whereby participants from several laboratories travel to one location to be trained together.

**Objectives:**

We assessed the effectiveness and cost of conducting SLMTA training in a decentralised manner as compared to centralised training.

**Methods:**

SLMTA was implemented in five pilot laboratories in Cameroon between October 2010 and October 2012 by means of a series of workshops, laboratory improvement projects and on-site mentorship. The first workshop was conducted in the traditional centralised approach. The second and third workshops were decentralised, delivered on-site at each of the five enrolled laboratories. Progress was monitored by repeated audits using the Stepwise Laboratory Quality Improvement Process Towards Accreditation (SLIPTA) checklist.

**Results:**

Audit scores for all laboratories improved steadily through the course of the programme. Median improvement was 11 percentage points after the first (centralised) training and an additional 24 percentage points after the second (decentralised) training. The estimated per-laboratory cost of the two training models was approximately the same at US$21 000. However, in the decentralised model approximately five times as many staff members were trained, although it also required five times the amount of trainer time.

**Conclusion:**

Decentralised SLMTA training was effective in improving laboratory quality and should be considered as an alternative to centralised training.

## Introduction

International Organization for Standardization (ISO) 15189 accreditation is viewed worldwide as the gold-standard mark of competence for clinical laboratories. However, the process of achieving international accreditation is labour-intensive, complex and expensive, making it challenging even for the best-resourced laboratories.^1^ These difficulties are magnified in resource-limited settings, where laboratories struggle to maintain staff levels and competence, basic infrastructure, equipment and supplies.^1,2,3,4,5^ As a result, few laboratories in sub-Saharan Africa are accredited and no laboratory in Cameroon has been accredited to international standards.^6^

Large-scale public health programmes, such as the US President’s Emergency Plan for AIDS Relief (PEPFAR), have highlighted gaps in laboratory services, emphasising the urgent need for quality improvement.^7^ The World Health Organization’s Regional Office for Africa (WHO AFRO) has responded by launching the Stepwise Laboratory Quality Improvement Process Towards Accreditation (SLIPTA) scheme, which is a phased approach to quality improvement.^8^

Training and mentoring in laboratory management have been identified as being critical for the implementation of quality management systems (QMS).^9^ However, many training programmes fail to result in measurable changes in laboratory practices because they focus more on theory and generic management topics than on practical aspects that can lead to direct implementation. They also lack follow-up with trainees to assist with application of knowledge into practice.^10^ The Strengthening Laboratory Management Toward Accreditation (SLMTA) programme is an innovative, task- and competency-based training programme that aims to address deficiencies in laboratory quality through a series of workshops, improvement projects and mentoring.^11^

The SLMTA three-workshop series is typically conducted in a central location that is logistically convenient for all laboratories in the training cohort. Centralised training allows many laboratories to be trained simultaneously and provides opportunities for laboratory networking and inter-facility knowledge-sharing. However, a centralised model can be expensive because of venue hiring and participant travel. Some have argued that decentralised training can be more sensitive to the needs of the trainees and tied to specific organisational or project goals, as trainers are able to respond rapidly to the needs of the audience and revise the training approach based on their feedback.^12^ In addition, decentralised training has been shown to improve relationships between local and central authorities and to increase institutional capacity.^13,14^

In an effort to improve laboratory quality, Cameroon began SLMTA implementation in 2010, with a first cohort of five laboratories. The initial training workshop was conducted in a centralised location. For the remaining two workshops, the programme shifted to a decentralised model, with facility-based training. The objective of this study is to compare the results and cost of decentralised training versus centralised training for the establishment of a QMS in five laboratories in Cameroon.

## Research methods and design

### Selection of laboratories

In 2009, four public hospital laboratories were selected by the Ministry of Public Health, Cameroon to enrol in the SLMTA programme: Buea Regional Hospital Laboratory (BuRHL), Bamenda Regional Hospital Laboratory (BRHL), Laquintinie Hospital Laboratory Douala (LHLD) and the Yaoundé Central Hospital Laboratory (YCHL). In August 2010, a private laboratory, Laboratoire d’Analyses Médicales du Centre (LAMC), was added to the four selected public laboratories in order to improve the link between the public and private sectors, which is essential for building sustainable national laboratory systems in resource-limited countries^3^ (Table 1).

**TABLE 1:** Laboratories included in Cameroon’s first cohort of the Strengthening Laboratory Management Toward Accreditation programme.

Laboratory name	Features^*^				
	Level	Type	Number of staff	Number of sections	Number of tests performed per week
BuRHL	Regional	Public	30	8	1300
BRHL	Regional	Public	33	7	1500
LHLD	Regional	Public	38	6	1200
YCHL	Regional	Public	42	4	1060
LAMC	Regional	Private	21	5	600

* Information provided by directors of the various laboratories.

BuRHL, Buea Regional Hospital Laboratory; BRHL, Bamenda Regional Hospital Laboratory; LHLD, Laquintinie Hospital Laboratory Douala; YCHL, Yaoundé Central Hospital Laboratory; LAMC, Laboratoire d’Analyses Médicales du Centre.

### Preparation

Global Health Systems Solutions (GHSS) – a local implementing partner – and the Cameroon office of the US Centers for Disease Control and Prevention (CDC) led SLMTA implementation. In preparation, all technical staff members from GHSS and the laboratory team of CDC-Cameroon underwent several training courses: (1) Good Clinical Laboratory Practice, provided by the South African National Accreditation System (SANAS); (2) use of ISO 15189 in internal audits and laboratory assessments toward accreditation, provided by SANAS; (3) SLMTA training, provided by CDC-Cameroon staff; and (4) laboratory mentorship, provided by a Clinton Health Access Initiative (CHAI) mentor.

To build capacity at the five selected SLMTA laboratories and to ensure sustainability, two employees from each laboratory were appointed as on-site mentors and trained for five days by a CHAI mentor on QMS, mentoring techniques, the 12 Quality System Essentials (QSEs), ISO 15189 and conducting laboratory audits using the SLIPTA checklist.

### SLMTA implementation and supplemental training

In October 2010, five participants from each of the five selected laboratories travelled to a central location in Mutengene, South West Region, Cameroon, for the first five-day SLMTA training workshop. The second and third trainings of five days each were held on-site in each laboratory, from February to March 2011 and June to July 2011, respectively. A catch-up training was provided to personnel who missed the initial centralised training. The number of personnel trained per site ranged from 12 to 24 persons, including laboratory managers and clinicians.

In addition to SLMTA training, the following centralised supplemental training courses were conducted for two employees from each laboratory: laboratory biosafety and biosecurity; development of standard operating procedures; internal audit; and use of a basic laboratory information system. Most of these supplemental trainings were done at the end of the three SLMTA training workshops.

A mentorship model that embeds a mentor within the daily routine of a laboratory for an extended period with a defined engagement schedule was used in these laboratories. These embedded mentors provided on-site coaching and guided the laboratories toward international accreditation by ensuring the implementation of improvement projects. In addition to embedded mentors, visiting mentors conducted two site visits following each workshop. Laboratory improvement projects are an integral part of the SLMTA programme, and were assigned to participants after each workshop. In subsequent workshops, participants presented their improvement projects and shared results and lessons learned. These sessions offered an opportunity for participants to learn from each other and facilitated the formation of a peer-learning network.

### Evaluation

The SLIPTA checklist was used to evaluate the laboratories’ progress, strengths and weaknesses. This checklist contains 12 sections (a total of 111 items) for a total of 258 points.^15^ SLIPTA checklist scores are categorised into star levels, with < 55% corresponding to zero stars, 55% – 64% one star, 65% – 74% two stars, 75% – 84% three stars, 85% – 94% four stars and 95% – 100% five stars. A GHSS staff member trained by WHO AFRO as an auditor conducted baseline audits of the four public laboratories between November and December 2009, and the fifth, private laboratory in August 2010. WHO AFRO-trained in-country auditors conducted four intermediate audits, just before the second and third SLMTA training workshops and after the third SLMTA workshop, in order to evaluate progress, identify gaps and develop action plans to close existing gaps.

Costs in US dollars to implement SLMTA training workshops were estimated for the centralised and decentralised models. For centralised training, we assumed that five people per laboratory would attend the three workshops. For decentralised training, we assumed that 24 participants would attend each on-site workshop. For both models, we assumed four trainers would be needed and that each workshop would last five days. Costs included lodging, per diem, land transport to the training venue, training materials for all participants, food and venue hiring (for centralised training only). We did not include salary or time missed from work for participants or trainers, nor other components of SLMTA implementation such as improvement projects, mentorship and audits, which would not be affected by training location. Trainer days were estimated for each model based on one travel day and five training days per workshop.

## Results

**FIGURE 1:**
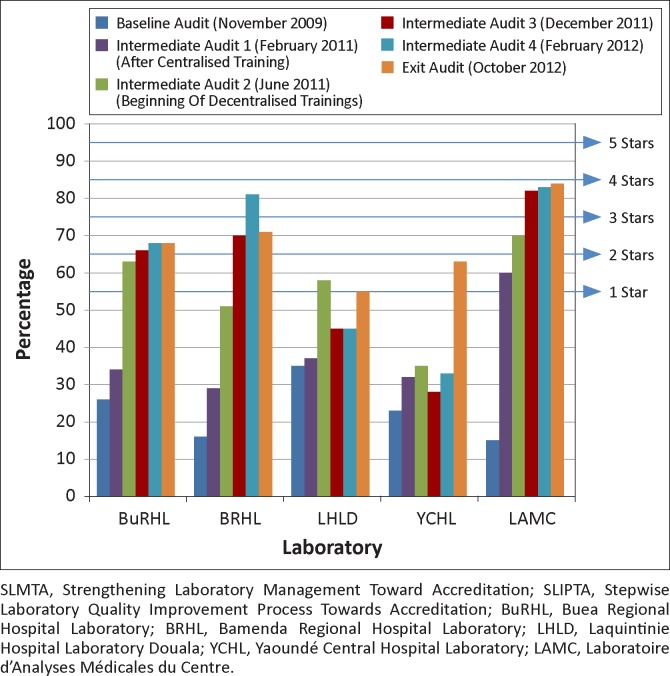
Performance of five Cameroon laboratories over 24 months of SLMTA implementation as measured by the SLIPTA checklist.

At baseline audit, the five laboratories scored a median of 23%, all at zero stars. Median scores increased steadily to 34% at the first intermediate audit, 58% at the second, 66% at the third and 68% at the fourth; they remained at 68% for the exit audit. Thus, there was a median total improvement of 45 percentage points. After 24 months of the SLMTA programme, two laboratories attained one star, two attained two stars and one attained three stars based on the audit scores. LAMC had the largest improvement of 69 percentage points (Figure 1).

The median improvement from the first SLMTA training to the second (intermediate audits 1 and 2) was 11 percentage points. After the first decentralised training, median scores improved an additional 24 percentage points. From the final training to exit, median scores improved 10 more percentage points. There was substantial variability in the timing of improvements. For example, LAMC had their greatest improvement between the baseline and first intermediate audit, whilst YCHL had their greatest improvement between the second intermediate audit and exit; LHLD’s score decreased slightly from the second intermediate audit to exit (Figure 1).

**FIGURE 2:**
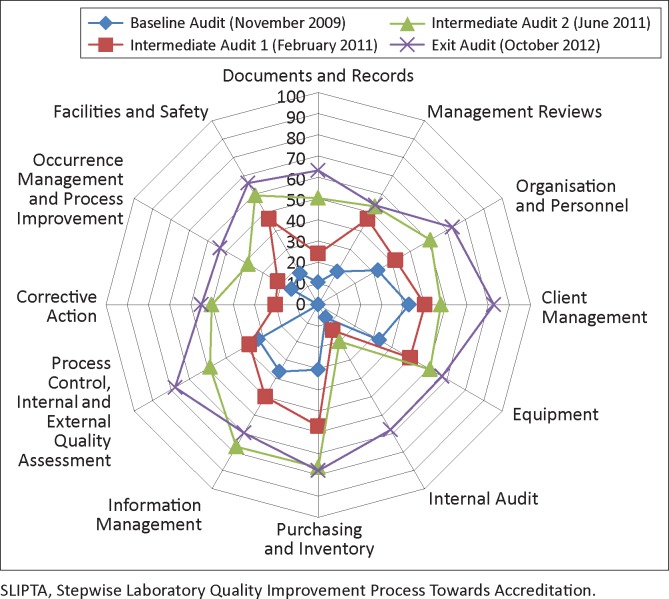
Average performance of the five laboratories measured at baseline, first and second intermediate, and exit audits. Average marks are expressed as a percentage of the total for each of the 12 Quality System Essentials sections of the SLIPTA checklist.

All five laboratories improved their scores in each of the 12 QSEs. Internal audit had the highest percentage average improvement (61 percentage points), followed by corrective action (55 percentage points) and documents and records (53 percentage points). Improvements from the baseline audit for five of the QSEs were greatest after the first SLMTA training, whilst seven of the QSEs improved most after the second SLMTA training (Figure 2).

The estimated cost of the workshop portion of SLMTA implementation for the two models is presented in Table 2. With the centralised training model, it would cost approximately $105 610 to hold the three SLMTA workshops for 25 participants from five laboratories ($21 122 per laboratory). If the workshops were decentralised and conducted in-house in each laboratory, the total cost would be approximately $107 400 to train 120 participants from the five laboratories ($21 480 per laboratory). Centralised training would require 72 trainer days; decentralised training would require 360 trainer days.

**TABLE 2:** Estimated cost in US dollars (USD) of conducting centralised versus decentralised SLMTA training workshops.

Assumptions		Centralised training		Decentralised training	
Number of laboratories		5		5	
Number of participants per laboratory		5		24	
Total number of participants		25		120	
Number of trainers		4		4	
Number of workshops		3		15	
Number of days per workshop^*^		5		5	

**Cost estimates**	**Unit cost (USD)**	**Cost for participants (USD)**	**Cost for trainers (USD)**	**Cost for participants (USD)**	**Cost for trainers (USD)**

Lodging	80/night/person	36 000	5760	-	28 800
Per diem	114/day/person	42 750	6840	-	34 200
Land transport	30/trip	2250	360	-	1800
Training materials	130/participant	3250	-	15 600	-
Cost of meeting	1800/training	5400	-	27 000	-
Hiring of venue	200/day	3000	-	-	-
Sub-total		92 650	12 960	42 600	64 800

**Grand Total (Participants plus Trainers)**		**105 610**		**107 400**	
**Cost per laboratory**		**21 122**		**21 480**	
**Cost per participant**		**4225**		**895**	

SLMTA, Strengthening Laboratory Management Toward Accreditation.

* Plus one travel day.

## Discussion

Laboratory scores improved steadily throughout the two-year programme, with all laboratories reaching at least the one-star level. Improvements after the decentralised second SLMTA workshop were twice as large as those after the centralised first workshop. The total estimated cost of the centralised and decentralised training models was about the same. However, in the decentralised model, approximately five times as many staff were trained as compared with the centralised model, whilst the centralised model required one-fifth the amount of trainer time as the decentralised model.

Decentralised workshops allowed more staff to participate in the training, facilitating shared understanding of the importance of quality improvement and the plan to achieve it. Hospital managers and clinicians were able to participate in the training alongside laboratory managers, improving clinician–laboratory interactions and providing them an opportunity to understand the potential for long-term improvement. On-site training enabled the use of familiar facilities to conduct interactive activities; SLMTA concepts could easily be shared amongst laboratory staff and any site-specific non-compliance could be discussed during the workshops. Finally, on-site workshops allowed the course to be tailored to the needs of the individual laboratories, with all workshop discussions related to site-specific challenges and solutions. On the other hand, centralised training fosters communication between laboratories, helping to build important networks. Participants in centralised training can learn from the experiences of other laboratories and get feedback on what did and did not work for them.

Two critical aspects to consider when implementing SLMTA are cost and manpower. In this study, we found that centralised and decentralised training cost roughly the same amount, at approximately $21 000 per laboratory. Savings made in the decentralised model for reduced costs for per diem, lodging, transport and venue hire were offset by the increased cost of trainers, training materials and food for the expanded group of participants. However, the two models have important consequences regarding manpower. Experience from other countries implementing SLMTA has suggested that staff attrition, especially through reassignment to other laboratories within the Ministry of Health system or to employment in private laboratories, is one of the critical challenges facing sustainability of results after SLMTA completion.^16^ When only a few staff members from each laboratory are trained, their departure has a pronounced effect on institutional memory and new staff must receive intensive training in order to continue the QMS work. In the decentralised model, the majority of laboratory staff are trained to implement QMS, reducing the impact of attrition of a few trained staff members. On the other hand, decentralised training requires far more trainer time, as the full series of workshops is conducted at each laboratory. The shortage of qualified trainers throughout Africa has been well noted.^17^ Usually, countries use trainers who are borrowed from their normal laboratory duties for the SLMTA training weeks. But when those weeks increase geometrically from three per cohort to three per laboratory, the feasibility of borrowing trainers is questionable. Globally, the median cohort size for SLMTA training has been 10 laboratories, with cohorts ranging from one to 27 laboratories.^18^ The logistical and manpower issues associated with decentralised training could quickly escalate in larger programmes, such that national laboratory programmes may need to consider adding staff dedicated to implementing SLMTA if decentralised training is desired.

Several challenges were faced in the implementation of the SLMTA programme in Cameroon. The first challenge was that governmental bureaucracy caused delays in project implementation. This problem was exacerbated by the lack of a National Laboratory Strategic Plan to define overall goals and stakeholders. This plan has now been developed and is pending adoption. Another major challenge was the lack of personnel trained and skilled in quality laboratory practices in selected facilities and insufficient numbers of local SLMTA trainers. This challenge is being resolved by recent training of three more SLMTA trainers and a plan to conduct in-country SLMTA training-of-trainers in the near future. The concept of QMS was entirely new to most laboratory staff in the selected facilities where a culture of quality has been lacking. As the staff undertook the training modules and understood the benefits of quality improvement, they became more cooperative and committed. Finally, in a system without biomedical engineers, there were difficulties with equipment maintenance. Most of the laboratory equipment is not available in the Cameroonian markets; this, coupled with the high cost of import duties in Cameroon, made equipment procurement and maintenance very costly. This challenge is being addressed with an on-going improvement project on equipment maintenance and calibration.

### Limitations of the study

Whilst the greater median improvement in scores after decentralised training suggests that it may have been more effective than centralised training, these results should be viewed in light of study limitations. Most importantly, this was an observational study of the natural progress of a programme; thus, there were no control laboratories on which to base a comparison. The difference in changes over time could be as a result of several factors, including timing of specific improvement projects undertaken after each workshop and variability in mentorship support. Furthermore, the pattern was not consistent amongst all five laboratories in the cohort; whilst three laboratories improved more after the second training than after the first, LAMC had its greatest improvement after the first and YCHL after the third. The immediate improvement in LAMC could possibly be because it is a private laboratory with few administrative bottlenecks that are common in larger public health facilities. Additional operational studies randomising cohorts to centralised versus decentralised training would provide more solid evidence of the relative effectiveness of these strategies.

### Conclusion

Quality laboratory systems are essential for providing patient care and global health. A competency-based programme such as SLMTA can assist public health laboratories in resource-limited settings to improve the quality of their services. The success of any programme depends on its sustainability. The lack of a national laboratory strategic plan, along with inadequate government funds and the absence of policies for equipment procurement and maintenance were major challenges faced by laboratories in Cameroon and other resource-limited settings and may continue even in the post-accreditation period. Training of facility-based mentors will help ensure continuous quality improvement, sustainability and country ownership. Although the challenges were many, SLMTA implementation successfully improved laboratory quality, ensuring better laboratory services and patient care.

Whether to conduct SLMTA trainings using a centralised or decentralised model will depend on situation-specific factors; however, decentralised training should be considered to widen the reach of the training within the laboratories. Cameroon intends to use decentralised training for future SLMTA cohorts.
